# Signal Processing and Machine Learning for Smart Sensing Applications

**DOI:** 10.3390/s23031445

**Published:** 2023-01-28

**Authors:** Ying-Ren Chien, Mu Zhou, Ao Peng, Ni Zhu, Joaquín Torres-Sospedra

**Affiliations:** 1 Department of Electrical Engineering, National Ilan University, Yilan 26047, Taiwan; 2 Chongqing Key Lab of Mobile Communications Technology, School of Communication and Information Engineering, Chongqing University of Posts and Telecommunications, Chongqing 400065, China; 3 School of Informatics, Xiamen University, Xiamen 361005, China; 4 AME-GEOLOC, University Gustave Eiffel, F-44344 Bouguenais, France; 5 ALGORITMI Research Centre, Universidade do Minho, 4800-058 Guimarães, Portugal

The Special Issue “Signal Processing and Machine Learning for Smart Sensing Applications” focused on the publication of advanced signal processing methods by means of state-of-the-art machine learning technologies for smart sensing applications. It targeted research areas that included radio navigation, indoor/outdoor positioning, mm-wave sensing, speech denoising, and noise cancellation, among many others. A secondary objective was to promote interdisciplinary collaborations between researchers in the fields of signal processing and machine learning technologies for smart sensing applications.

A total of 17 works were published within this Special Issue, where we can find works that are dealing with the more cutting-edge solutions for audio filtering for speech enhancement, identification and mitigation of some types of jamming, electroencephalogram processing for sleep-arousal detection, localization using magnetic field information, processing direction-of-arrival, detection of defects, fall detection, tracing healthcare data in real-time, as well as learn how signals propagate under non-line-of-sight conditions. The main contributions are briefly described in the remainder of this editorial.

Zhou et al. [1] proposed a new algorithm using bone-conduction (BC) signals to assist dual-microphone generalized sidelobe canceller (GSC) adaptive beamforming for speech enhancement. First, the BC signals were used to conduct highly reliable voice activity detection (VAD), assisted adaptive noise canceller (ANC), and adaptive block matrix (ABM) weight coefficient updates in GSC. Second, an adaptive compensation filter (CF) was designed to compensate the amplitude and phase difference between air-conduction (AC) and BC signals. Third, wind noise was detected and replaced with the output of CF to recover low-frequency speech components from the wind noise. Finally, a real-time neural network-based postfilter was designed and trained to effectively remove the residual noise. Experimental results showed that the proposed algorithm effectively improves signal-to-noise ratio (SNR) and speech quality in different scenarios, and the assistance of BC signals can effectively improve the noise reduction performance of beamforming.

Qin et al. [2] introduced the minimum description length (MDL) principle and atomic norm into the field of low-rank matrix recovery and proposed a novel non-parametric low-rank matrix approximation method called MDLAN. The existing algorithms had difficulty tackling the proposed optimization problem; thus, the authors considered an approximation of the original problem. Their method selected the best atoms to search for the best approximation of the low-rank matrix, and it also could find sparse noise simultaneously. The experimental setup included a comparison with state-of-the-art methods using synthetic data and three real sensing low-rank applications, i.e., HDR imaging, background modelling based on a video sensor and the removal of noise and shadows from face images. The experimental results using the synthetic and real sensing datasets demonstrated the effectiveness and robustness of the proposed approach.

Lan et al. [3] proposed a method for identifying deceptive jamming of chirp radar. Firstly, the short-time Fourier transform (STFT) time-frequency diagram of the jamming signal was used as the input of the jamming identification network, which provided the time-frequency information of the jamming signal for the identification network, and achieved the identification of some types of jamming. Secondly, adding the time-frequency diagram of the echo signal to the network input provided information about the real signal and the difference between the jamming and the real signal for identification by the network, which increased the number of types of jamming that the network recognized. Thirdly, the fusion of multiple-pulse jamming plus echo time-frequency graphs was used as the input of the network, which provided jamming pulse information for the identification network and achieved the identification of all interference types. Finally, by replacing the time-frequency diagram of the echo signal with the time-frequency diagram of the original signal as a part of the network input, the accuracy rate of interference recognition increased under low jamming-to-noise ratios (JNR) conditions. By building a model with the ResNet50 network as the core, the simulation tested the effectiveness of the jamming identification method and the improved method.

Zhao et al. [4] a more systematic method for extracting and evaluating important pressure features, demonstrating the feasibility of using fewer pressure sensors for driver posture monitoring and suggesting research directions for better sensor designs. As opposed to the use of the 15 important features by the single RF classifier in [5], this work selected 40, 24, and 22 important features to classify trunk postures, left-foot postures, and right-foot postures, respectively. The authors applied five different supervised machine-learning techniques to recognize the postures of each body part and used leave-one-out cross-validation to evaluate their performance. A uniform sampling method was used to reduce the number of pressure sensors, and five new layouts were tested by using the best classifier. Results showed that the random forest classifier outperformed the other classifiers with an average classification accuracy of 86% using the original pressure mats and 85% when only 8% of the pressure sensors were available.

Chien et al. [6] proposed a novel sleep-arousal detection method that requires only single-lead electroencephalogram (EEG) signals based on the stacking ensemble learning framework. The meta-classifier stacks four sub-models: (1) 1D convolutional neural network (CNN); (2) recurrent neural network (multi-layer LSTM); (3) merged 1D CNN and RNN; and (4) random forest algorithm. First, the 1D CNN network extracts the embedded features in the time-domain waveform. Second, the RNN learns the temporal dependence in the band power and power ratio features. Third, the merged 1D CNN and RNN networks extract complementary missing information for either CNN or RNN alone. Finally, the random forest algorithm exploits the expert-defined features calculated from both time and frequency domains. The authors verified the effectiveness of the proposed method using the open-accessed database compiled in [7]. The improvements of the meta-classifier over any sub-model can be up to 9.29%, 7.79%, 11.03%, 8.61%, and 9.04%, respectively, in terms of specificity, sensitivity, precision, accuracy, and AUROC.

Kaneko et al. [8] introduced a method for observing microvascular waves (MVW) by extracting different images from the available images in the video taken with consumer cameras. Microvascular vasomotion is a dynamic phenomenon that can fluctuate over time for a variety of reasons and its sensing is used for a variety of purposes. The special device, a side stream dark field camera (SDF camera) was developed in 2015 for the medical purpose to observe blood flow from above the epidermis. However, without using SDF cameras, smart signal processing can be combined with a consumer camera to analyze the global motion of microvascular vasomotion. MVW is a propagation pattern of microvascular vasomotions which reflects the biological properties of the vascular network. In addition, even without SDF cameras, MVW can be analyzed as a spatial and temporal pattern of microvascular vasomotion using a combination of advanced signal processing with consumer cameras. In this paper, the authors demonstrated that such vascular movements and MVW can be observed using consumer cameras, showing a classification using it.

Liu et al. [9] developed a two-level framework for jamming decision-making against the adaptive radar and proposed a dual Q-learning model to optimize the jamming strategy. The jamming mode and pulse parameters were determined hierarchically, greatly reducing the dimensionality of the search space and improving the learning efficiency of the model. In addition, the authors proposed a new method to calculate the jamming effectiveness by measuring the distance of indicator vectors, where the indicators are dynamically weighted to adapt to the changing environment. The jamming effectiveness evaluation result is served as the feedback value to update the dual Q-learning model. Simulation results proved that the proposed solution minimised the radar’s threat level. Furthermore, they compared it with standard Q-learning. Their proposed method improved the average jamming-to-signal ratio (JSR) by 4.05% and reduces the convergence time by 34.94%.

Sasaki [10] presented a study where they demonstrated that machine learning is suitable for solving inverse problems present in localization systems based on magnetic field information. The authors put emphasis on comparing performances obtained with k-Nearest Neighbors (k-NN) and Artificial Neural Networks (ANNs) that were adopted for machine learning. The authors numerically evaluated the accuracy of the target positions predicted by k-NN and ANNs by considering the 2 m × 2 m × 2 m cubic space. Prob(d<0.1 m) and $d_{av}$ obtained with k-NN were 84% and 66 mm, respectively, and those obtained with ANNs were 97% and 44 mm, respectively. Despite taking longer to train, ANNs were superior to k-NN in terms of localization accuracy. The k-NN was still valid for predicting fairly accurate target positions within limited training times.

Tian et al. [11] proposed a novel algorithm that extends the underdetermined direction-of-arrival (DOA) estimation in a Sparse Circular Array (SCA), and an inverse beamspace transformation combined with the Gridless SPICE (GLS) algorithm to complete the covariance matrix sampled by SCA. The DOAs are then obtained by solving a polynomial equation with using the Root-MUSIC algorithm. The proposed algorithm is named GSCA. Monte-Carlo simulations are performed to evaluate the GSCA algorithm, the spatial spectrum plots and RMSE curves demonstrated that the GSCA algorithm can give reasonable results of underdetermined DOA estimation in SCA. Meanwhile, the performance of the algorithm under various configurations of SCA is also evaluated. Numerical results indicated that the GSCA algorithm can provide access to solve the DOA estimation problem in Uniform Circular Array (UCA) when random sensor failures occur.

Bačnar et al. [12] elaborated on and compares three classes of time-frequency representations (TFRs): Cohen’s, affine, and reassigned, including the theoretical background of the selected TFRs belonging to these classes. Next, the authors performed extensive numerical simulations on non-stationary signals, including both synthetic and real-life examples. The methods were applied both on synthetic and real-life non-stationary signals. The obtained results were assessed with respect to time-frequency concentration (measured by the Rényi entropy), instantaneous frequency (IF) estimation accuracy, cross-term presence in the TFRs, and the computational cost of the TFRs. This study gives valuable insight into the advantages and limitations of the analyzed TFRs and assists in selecting the proper distribution when analyzing given non-stationary signals in the time-frequency domain.

Mei et al. [13] studied the two-dimensional direction of arrival (2D-DOA) estimation problem in a switching uniform circular array (SUCA), and proposed a covariance matrix completion algorithm for 2D-DOA estimation in a SUCA. The proposed algorithm estimated the complete covariance matrix of a fully sampled UCA (FUCA) from the sample covariance matrix of the SUCA through a neural network. Afterwards, the MUSIC algorithm was performed for 2D-DOA estimation with the completed covariance matrix. The authors conducted Monte Carlo simulations to evaluate the performance of the proposed algorithm in various scenarios; the performance of 2D-DOA estimation in the SUCA gradually approached that in the FUCA as the SNR or the number of snapshots increases, which means that the advantages of a FUCA can be preserved with fewer RF chains. In addition, the proposed algorithm is able to implement the underdetermined 2D-DOA estimation.

Lin et al. [14] proposed a deep learning-based framework named SigdetNet, which takes the power spectrum as the network’s input to localize the spectral locations of the signals. In the proposed framework, Welch’s periodogram was applied to reduce the variance in the power spectral density, followed by logarithmic transformation for signal enhancement. In particular, an encoder–decoder network with the embedding pyramid pooling module was constructed, aiming to extract multi-scale features relevant to signal detection. The influence of the frequency resolution, network architecture, and loss function on the detection performance was investigated. Extensive simulations were carried out to demonstrate that the proposed multi-signal detection method can achieve better performance than several representative benchmark methods, including the localization algorithm based on double-thresholding (LAD) and the full convolutional network (FCN).

Mao et al. [15] studied the eye-in-hand architecture in conjunction with deep learning and convolutional neural networks to automate the detection of defects in forged aluminium rims for electric vehicles. RobotStudio software was used to simulate the environment and path trajectory for a camera installed on an ABB robot arm to capture 3D images of the rims. Four types of surface defects were examined: (1) dirt spots, (2) paint stains, (3) scratches, and (4) dents. Generative adversarial networks (GAN) and deep convolutional generative adversarial networks (DCGAN) were used to generate additional images to expand the depth of the training dataset. The authors also developed a graphical user interface and software system to mark patterns associated with defects in the images. The defect detection algorithm based on YOLO algorithms made it possible to obtain results more quickly and with higher mean average precision (mAP) than that of existing methods. Experiment results demonstrated the accuracy and efficiency of the proposed system, which was shown to be a helpful rim defective detection system for industrial applications. In particular, the proposed approach proved high efficiency in enhancing the accuracy, recall, and precision ratios of YOLO v3, with an increase of +6.5%, and YOLO v4, with an increase of +37.7%.

Hsu et al. [16] proposed a GAN-like network for fall detection. The proposed network inspected the reconstruction quality of future frame sequences based on the subject’s motion, while the proposed framework was able to cope with the extreme scenario when the subjects were blocked or unseen after falling. In addition, to ensure users’ privacy, the depth images or the thermal images were used as the inputs, and several denoising schemes were proposed to prevent the noises in the input images from interfering with the prediction results. Accordingly, the unsupervised learning approach was adopted that considered fall accidents as anomalous events to tackle the problem of an imbalanced dataset from data acquisition. The proposed system took sequential frames as the inputs to predict future frames based on a GAN structure and provided (1) multi-subject detection, (2) real-time fall detection triggered by motion, (3) a solution to the situation that subjects were occluded after falling, and (4) a denoising scheme for depth images. Except for the advantages mentioned above, the experimental results on two public datasets showed that the proposed framework for fall detection achieves state-of-the-art performance.

Tuan et al. [17] developed a range of motion sensing system (ROMSS) to simulate the function of the elbow joint, with errors less than 0.76 degrees and 0.87 degrees in static and dynamic verification by the swinging and angle recognition modules, respectively. In the simulation process, the γ correlation coefficient of the Pearson difference between the ROMSS and the universal goniometer was 0.90, the standard deviations of the general goniometer measurements were between ±2 degrees and ±2.6 degrees, and the standard deviations between the ROMSS measurements were between ±0.5 degrees and ±1.6 degrees. With the ROMSS, a cloud database was also established; the data measured by the sensor could be uploaded to the cloud database in real-time to provide timely patient information for healthcare professionals. We also developed a mobile app for smartphones to enable patients and healthcare providers to easily trace the data in real time. Historical datasets with joint activity angles could be retrieved to observe the progress or effectiveness of disease recovery so the quality of care could be properly assessed and maintained.

Xiao et al. [18] studied the problem of robust beamforming for a high-speed moving array platform under the background of spatially correlated coloured noise. After analyzing the influence of the background noise on the adaptive beamforming, an adaptive wide null beamforming algorithm based on the Toeplitz matrix structure projection constraint was proposed. The subspace of the integration of the correlation matrix of the steering vector in the pre-determined extended region was first extracted, and the constraint matrix and the projection transformation matrix were constructed. The covariance matrix of the array-received signal with a Toeplitz structure was then constructed using the correlation numbers of the received data of each array element and the reference array element. Finally, the optimal weight vector of the array was obtained through a projection transformation and linear constraint on the constructed covariance matrix. The simulation results showed that the anti-jamming performance of the proposed algorithm was less affected by the background noise. It could perform the null broadening of the beam in the pre-determined region and solve the problems of distortion sidelobe lifting and main lobe offset of adaptive beamforming caused by spatially correlated colour noise.

Wang et al. [19] proposed the MT-GCNN (Multi-Task Gated Convolutional Neural Network), a novel multiple transmitters localization scheme based on deep multi-task learning to learn the non-line-of-sight (NLOS) propagation features and achieve localization. The multi-task learning network decomposed the problem into a coarse localization task and a fine correction task. In particular, the MT-GCNN used an improved gated convolution module to efficiently extract features from sparse sensing data. In the training stage, a joint loss function was proposed to optimize the two branches of tasks. In the testing stage, the MT-GCNN was able to predict the classified grids and corresponding biases jointly to improve the overall performance of localization. In the urban scenarios challenged by NLOS propagation and sparse deployment of sensors, numerical simulations demonstrated that the proposed MT-GCNN framework is more accurate and robust than other algorithms.

## Data Availability

The research discussed in this editorial is available in https://www.mdpi.com/journal/sensors/special_issues/spmlssa (accessed on 2 January 2023).
